# An Anodic Phase Can Facilitate Rather Than Weaken a Cathodic Phase to Activate Neurons in Biphasic-Pulse Axonal Stimulations

**DOI:** 10.3389/fnins.2022.823423

**Published:** 2022-03-17

**Authors:** Lvpiao Zheng, Zhouyan Feng, Yipeng Xu, Yue Yuan, Yifan Hu

**Affiliations:** Key Laboratory of Biomedical Engineering for the Ministry of Education, College of Biomedical Engineering and Instrument Science, Zhejiang University, Hangzhou, China

**Keywords:** biphasic-pulse stimulation, activation efficiency, antidromically-evoked population spike, pulse intensity, hyperpolarization block

## Abstract

Electrical pulses have been promisingly utilized in neural stimulations to treat various diseases. Usually, charge-balanced biphasic pulses are applied in the clinic to eliminate the possible side effects caused by charge accumulations. Because of its reversal action to the preceding cathodic phase, the subsequent anodic phase has been commonly considered to lower the activation efficiency of biphasic pulses. However, an anodic pulse itself can also activate axons with its “virtual cathode” effect. Therefore, we hypothesized that the anodic phase of a biphasic pulse could facilitate neuronal activation in some circumstances. To verify the hypothesis, we compared the activation efficiencies of cathodic pulse, biphasic pulse, and anodic pulse applied in both monopolar and bipolar modes in the axonal stimulation of alveus in rat hippocampal CA1 region *in vivo*. The antidromically evoked population spikes (APS) were recorded and used to evaluate the amount of integrated firing of pyramidal neurons induced by pulse stimulations. We also used a computational model to investigate the pulse effects on axons at various distances from the stimulation electrode. The experimental results showed that, with a small pulse intensity, a cathodic pulse recruited more neurons to fire than a biphasic pulse. However, the situation was reversed with an increased pulse intensity. In addition, setting an inter-phase gap of 100 μs was able to increase the activation efficiency of a biphasic pulse to exceed a cathodic pulse even with a relatively small pulse intensity. Furthermore, the latency of APS evoked by a cathodic pulse was always longer than that of APS evoked by a biphasic pulse, indicating different initial sites of the neuronal firing evoked by the different types of pulses. The computational results of axon modeling showed that the subsequent anodic phase was able to relieve the hyperpolarization block in the flanking regions generated by the preceding cathodic phase, thereby increasing rather than decreasing the activation efficiency of a biphasic pulse with a relatively great intensity. These results of both rat experiments and computational modeling firstly reveal a facilitation rather than an attenuation effect of the anodic phase on biphasic-pulse stimulations, which provides important information for designing electrical stimulations for neural therapies.

## Introduction

Electrical stimulations of pulse sequences have been applied extracellularly in the brain for treating neurological and psychiatric diseases (Krauss et al., 2021). For extracellular stimulations, a cathodic pulse can generate a current sink in the space immediately around the stimulation electrode, thereby generating an outward transmembrane current to depolarize the membranes of neuronal structures to activate neurons (Rubinstein et al., 2001). However, persistent stimulation of monophasic pulses can cause damage in brain tissues due to accumulations of electrical charges. The charge accumulations may result in an irreversible Faradaic reaction to produce toxic chemicals and to corrode electrodes (Merrill et al., 2005). Therefore, to eliminate the harmful side effects, charge-balanced biphasic pulses are commonly used by setting an anodic pulse (or anodic phase) immediately following a cathodic pulse (or cathodic phase) (Lilly et al., 1955; Merrill et al., 2005).

An anodic pulse applied extracellularly can itself generate a current source immediately around the stimulation electrode, thereby generating an inward transmembrane current to hyperpolarize the neuronal membranes. Thus, the subsequent anodic phase in a biphasic pulse is supposed to reverse the depolarization effect of the preceding cathodic phase and to elevate the threshold of the cathodic phase to activate neurons (Cappaert et al., 2012; Brocker and Grill, 2013). Techniques have been developed to decrease the reverse effect of the anodic phase, such as designing asymmetry waveforms of biphasic pulses (Macherey et al., 2010; Koivuniemi and Otto, 2011; Haji Ghaffari et al., 2020) and setting an inter-phase gap (IPG) between the cathodic phase and the anodic phase (Gorman and Mortimer, 1983; Weitz et al., 2014; Eickhoff and Jarvis, 2021). A short IPG of 100 μs has been shown to be sufficient to eliminate the reversal effect of the anodic phase without impairing its function to prevent the charge accumulations (Merrill et al., 2005).

On the other hand, a hyperpolarization current source is always accompanied by a depolarization current sink, *vice versa*, no matter what phase (or what type) of pulses is applied, cathodic or anodic pulses in monophase or biphase. In addition, the structures of a neuron are complex, including a soma, dendrites, and a long axon spreading in a relatively large space. Therefore, an anodic pulse generating a hyperpolarization at one site of a neuron can simultaneously generate a depolarization at other sites on the same neuron, especially along an axon. Consequently, an anodic pulse may also depolarize a neuron to fire an action potential (AP) at a certain site, the so-called virtual cathode in an axon (Brocker and Grill, 2013). Because the virtual cathode is at a distant location from the stimulation electrode, its current sink is relatively weak. With an identical stimulation intensity, the activation efficiency of an anodic pulse is much smaller than a cathodic pulse (Ranck, 1975; Tai et al., 2009; Brocker and Grill, 2013). Nevertheless, a preceding anodic phase may also activate neurons as an activate phase with a small and long subsequent cathodic phase for charge balance (McIntyre and Grill, 2000). Therefore, a biphasic pulse does not necessarily activate less neurons than a monophasic cathodic pulse since an anodic phase may exert a net effect of depolarization instead of hyperpolarization under some circumstances.

Many previous studies have compared the activation efficiencies of biphasic pulses with monophasic pulses. Some studies have shown that a cathodic pulse is more efficient than a biphasic pulse in activating individual neurons—for example, in stimulations of cat auditory nerves, the threshold to induce single-unit spikes is lower by a cathodic pulse than by a biphasic pulse (Miller et al., 2001). Computational modeling has also shown that a cathodic pulse has a lower activation threshold than both a biphasic pulse and an anodic pulse (Reilly et al., 1985). However, other studies have shown that, for neuronal populations, an anodic pulse has a lower threshold than a cathodic pulse in recruiting the responses of cortical neurons with suprachoroidal stimulation (Kanda et al., 2004; John et al., 2013) because the anodic pulse may depolarize neurons or neuronal elements distant from the stimulation electrode. Thus, neurons in different distances from the stimulation electrode could have various responses to different types of pulses, thereby generating a diverse integrated reaction of a neuronal population. We hypothesized here that the subsequent anodic phase could facilitate rather than weaken the activation effect of a biphasic pulse on a population of neurons.

To verify the hypothesis, we compared the integrated firing of a population of neurons responding to the axonal stimulation of three types of pulses—a cathodic pulse, an anodic pulse, and a biphasic pulse—in rat hippocampal CA1 region *in vivo*. The integrated firing of neuronal population was evaluated by the antidromically evoked population spike (APS) by taking advantage of the dense arrangement of somata and the clear lamellar organization of neuronal elements in the hippocampal region (Lipski, 1981). The reason that axonal stimulations were applied in this study is that axons are everywhere in the brain, and they have the lowest threshold to respond to pulse stimulations among the structure elements of a neuron (Nowak and Bullier, 1998; Rattay, 1999). A pulse tends to first activate the axons of neurons in extracellular stimulations in the brain. Thus, axonal responses play an important role in neural stimulation therapies (Gradinaru et al., 2009). In addition, we also used a computational model to reveal the possible underlying mechanisms of axonal stimulations to explain the observations of the animal experiments. The results of this study provide both experimental and theoretical information of neuronal activations by various pulses, which may guide the development of neural stimulation paradigms.

## Materials and Methods

### Animal Experiments

#### Animal Surgeries

**FIGURE 1:**
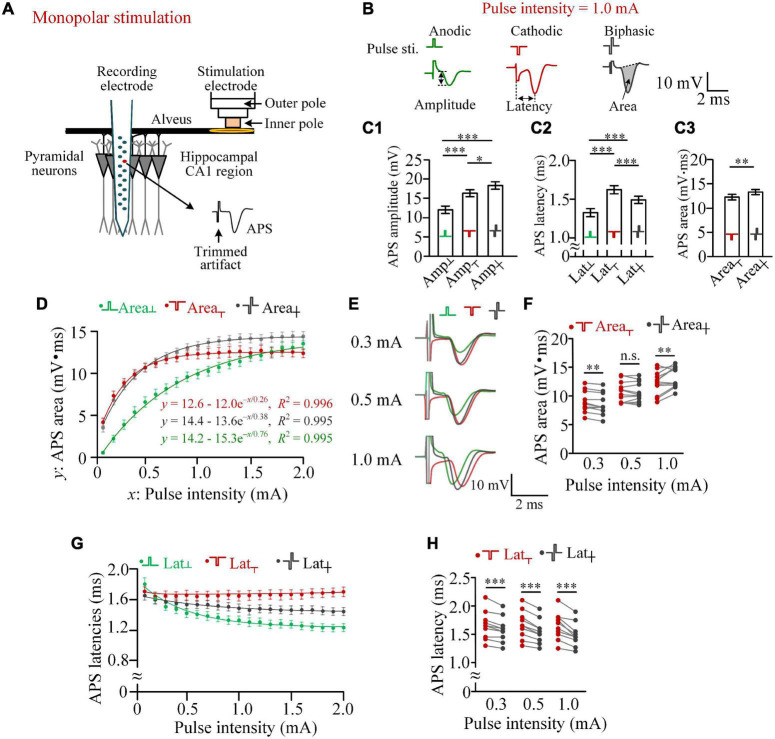
Differences in the activation efficiencies of anodic, cathodic, and biphasic pulses in monopolar stimulations in rat hippocampal CA1 region. **(A)** Schematic diagram of the locations of recording and stimulation electrodes. **(B)** Examples of antidromically evoked population spike (APS) potential waveforms evoked by the three types of pulses with an identical pulse intensity of 1.0 mA, together with the definitions of APS amplitude, APS latency, and APS area. **(C)** C1 and C2: comparisons of the amplitudes and latencies of APS evoked by the three types of pulses with a pulse intensity of 1.0 mA (*p < 0.05, ^***^p < 0.001, two-way ANOVA, *post hoc* least significant difference test, *n* = 12). C3: comparisons of the areas of APS evoked by cathodic and biphasic pulses (^**^p < 0.01, paired *t*-test, *n* = 12). **(D)** Changes of mean APS areas evoked by the three types of pulses against the pulse intensity increasing from 0.1 to 2.0 mA with a step of 0.1 mA, together with exponential curve fitting. **(E)** Examples of superimposed APS waveforms evoked by the three types of pulses with pulse intensities of 0.3, 0.5, and 1.0 mA. **(F)** Comparisons of areas of APS evoked by cathodic and biphasic pulses with pulse intensities of 0.3, 0.5, and 1.0 mA (^**^p < 0.01; n.s., not significant, paired *t*-test; *n* = 12). **(G)** Changes of mean APS latencies evoked by the three types of pulses against the pulse intensity. **(H)** Comparisons of latencies of APS evoked by cathodic and biphasic pulses with pulse intensities of 0.3, 0.5, and 1.0 mA (^***^p < 0.001, paired *t*-test, *n* = 12).

The animal experiment was approved by the Institutional Animal Care and Ethics Committee, Zhejiang University (ethics code: ZJU20210108). Forty-six adult Sprague–Dawley rats (250–400 g) were anesthetized by urethane (1.25 g/kg, i.p.), and experiments were performed in a stereotaxic apparatus (Stoelting Co., United States). The procedure of surgery and electrode implantation has been reported previously (Feng et al., 2014). In brief (Figure 1A), a concentric bipolar stimulation electrode (#CBCSG75, FHC Inc., United States; inner pole: Platinum/iridium, diameter 75 μm; outer pole: stainless steel, diameter 250 μm) was placed at the alveus of CA1 region to activate the axons of CA1 pyramidal neurons. A recording electrode of 16-channel array (#Poly2, NeuroNexus Technologies Inc., United States) was placed in the hippocampal CA1 region where the neurons were activated antidromically by stimulations. Unit spikes of spontaneous firing of neurons and the waveforms of APS recorded along the electrode channels were used to justify the positions of both the stimulation and recording electrodes (Kloosterman et al., 2001; Gold et al., 2006; Leung and Peloquin, 2006). The positions of electrodes were kept fixed throughout the period of data collection. After the animal experiments, the correction of electrode positions was confirmed by a histology analysis of brain slices (Wang et al., 2021).

#### Recording and Stimulating

Raw signals collected by the recording electrode in the hippocampal CA1 region were amplified 100 times by an amplifier (Model 3600, A-M System Inc., United States) with a band-pass filtering range of 0.3–5,000 Hz. Then, the amplified signals were sampled by a PowerLab data acquisition system (Model PL3516, ADInstruments Inc., Australia) with a sampling rate of 20 kHz. The channel that recorded the largest APS and dense unit spikes was judged to be located in the layer of somata and used to extract APS data (Figure 1A).

The concentric bipolar electrode was used to deliver monopolar or bipolar stimulations. For monopolar stimulations, only the inner pole was used to deliver a stimulus current that returned from a stainless steel screw fixed on the rat’s skull over the cerebellum. For bipolar stimulations, both the inner and outer poles were used to deliver a stimulus current flowing in the limited space between the two poles.

The stimuli were constant-current pulses generated by a programmable stimulator (Model 3800, A-M System Inc., United States). Three types of pulses—cathodic pulse, anodic pulse, or symmetric biphasic pulse (anode following cathode with zero IPG)—were delivered with monopolar mode or bipolar mode. In addition, a paired pulse formed by inserting an IPG of 100 μs between the two phases of a biphasic pulse was also used. The polarity of the pulse phase was defined as the polarity of current flowing out of the inner pole of the concentric bipolar electrode. The width per pulse phase was 100 μs, and the current intensity of the pulse (i.e., the absolute intensity per phase) was in the range of 0.1–2.0 mA. Stimulation artifacts were trimmed in the illustrations for clarified APS waveforms.

#### Data Analysis

The characteristic values of APS potential waveforms have been used to evaluate the synchronous firing of a population of neurons, such as area and amplitude of APS representing the number of firing neurons (Andersen et al., 1971; Richardson et al., 1987; Varona et al., 2000; Franco et al., 2016). Here the amplitude, area, and latency of the APS waveform were calculated to evaluate the differences in neuronal firing induced by different types of pulses (Figure 1B). For accuracy, each value was the average of three successive APSs evoked repeatedly with an interval of 5 s. The APS amplitude was measured as the potential difference of its falling phase. The APS area was measured as the integration of the area surrounded by the APS curve and a tangent line covering its opening (Theoret et al., 1984). The APS latency was the time distance between the onset of the stimulus pulse and the negative peak of APS.

All statistical data were shown as mean ± standard error of mean (SEM), with *n* representing the number of rats. Two-way ANOVA with *post hoc* least significant difference (LSD) test or paired *t*-test was used to judge the significance of differences among or between data groups. Exponential curve fitting was used to describe the changes of APS area against the increase of pulse intensity. Coefficients of determination (*R*^2^) were used to evaluate the performance of the fitting curves.

### Computational Modeling

The alveus was modeled as a bundle of myelinated axons to reveal the mechanisms of neuronal responses to different types of axonal stimulations by using NEURON package version 7.5 (Hines and Carnevale, 1997) and COMSOL multiphysics 5.3 (COMSOL Inc., Sweden). The details of the computational model have been reported previously (Guo et al., 2018; Zheng et al., 2020).

Each axon was composed of 21 nodes (Node_0_ to Node_20_) on which sodium and potassium channels were distributed as previously reported (Guo et al., 2018). In addition, here we set the activation threshold of sodium channel as –46 mV because the threshold of pyramidal cells to generate APs can be in the region of –60 to –38 mV (Shah et al., 2008; Sørensen et al., 2017).

In the computational model, the stimulation electrode was located above the center node of the axon (i.e., Node_10_). Similar to the three types of pulses used in rat experiments, the monophasic pulse (cathode or anode) and the biphasic pulse with an intensity of 0.3 or 1.0 mA were applied in the modelings—that is, the potential values of electrical field generated by the pulses were calculated by the COMSOL and were then loaded into the NEURON model to apply on axons (Zheng et al., 2020).

The responses of five axons with different electrode-to-fiber distances of 25, 70, 170, 200, and 210 μm were collected. The changes of membrane potentials (*V*_*m*_) on each node of the axons were analyzed to judge whether or not a neuron successfully generated an action potential upon a pulse stimulation and on which node the action potential was initiated.

## Results

### The Anodic Phase of a Biphasic Pulse Can Facilitate the Activation of Neuronal Population in Certain Monopolar Stimulations

In monopolar stimulations, the pulse current flowed between the inner pole of the stimulation electrode at the alveus and the remote return electrode above the cerebellum. The current activated the neurons in the pathway, especially the alveus immediately under the stimulation electrode. The evoked firing in the axons of the alveus then traveled antidromically to the somata of CA1 pyramidal neurons and generated an APS collected by the recording electrode (Figure 1A).

**FIGURE 2:**
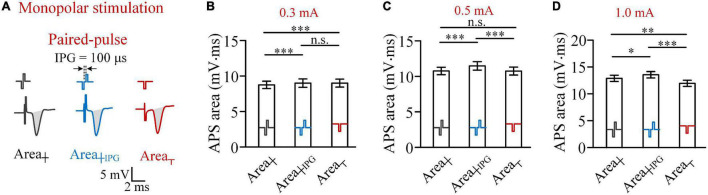
Comparisons of areas of APS evoked by biphasic pulses and paired pulses, with an inter-phase gap of 100 μs, and cathodic pulses in monopolar stimulations in rat experiments. **(A)** Examples of APS evoked by the three types of pulses. **(B–D)** Comparisons of mean APS areas evoked by the three types of pulses with pulse intensities of 0.3, 0.5, and 1.0 mA, respectively (*p < 0.05, ^**^p < 0.01, ^***^p < 0.001; n.s., not significant, two-way ANOVA, *post hoc* least significant difference test, *n* = 7).

With a pulse intensity of 1.0 mA, an anodic pulse was able to induce an obvious APS, indicating its activation effect of the virtual cathode (Figure 1B). Due to the relatively weak activation of the virtual cathode, the mean amplitude of APS evoked by an anodic pulse (Amp_┴_) was significantly smaller than the amplitudes of APS evoked by a cathodic pulse (Amp_┬_) or by a biphasic pulse (Amp_┼_) with the identical absolute intensity (1.0 mA) per phase. Surprisingly, the mean Amp_┼_ was significantly greater than the mean Amp_┬_ (Figure 1C-1), indicating a facilitating effect, not a reversing effect, of the anodic phase on the effect of the preceding cathodic phase. In addition, significant differences existed in APS latencies among the three types of pulses (Figure 1C-2). The latency of APS evoked by an anodic pulse was significantly shorter than those of APS evoked by the other two types of pulses, indicating different initial sites of the neuronal firing evoked by the different types of pulses.

**FIGURE 3:**
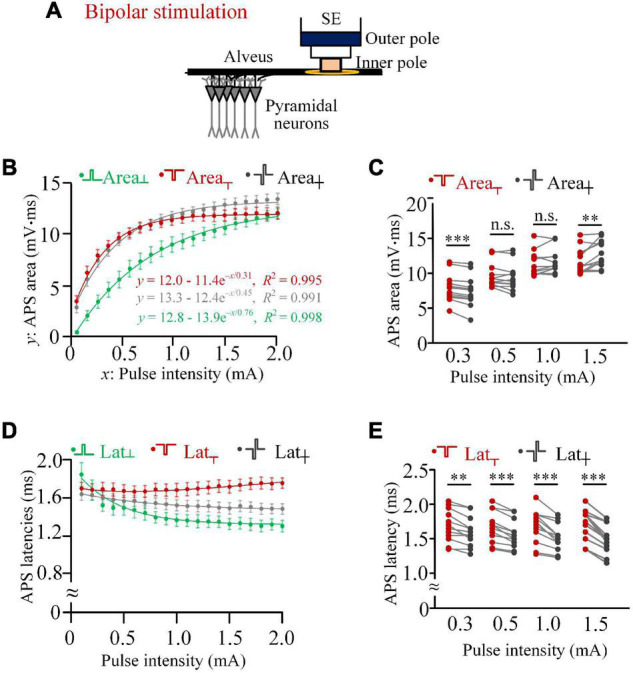
Comparisons of activation efficiencies of anodic, cathodic, and biphasic pulses in bipolar stimulations in rat experiments. **(A)** Schematic diagram of the bipolar stimulation. **(B)** Changes of mean areas of antidromically evoked population spike (APS) evoked by the three types of pulses against the pulse intensity, together with exponential curve fitting. **(C)** Comparisons of mean areas of APS evoked by cathodic and biphasic pulses with pulse intensities of 0.3, 0.5, 1.0, and 1.5 mA (^**^p < 0.01, ^***^p < 0.001; n.s., not significant, paired *t*-test, *n* = 12). **(D)** Changes of mean latencies of APS evoked by the three types of pulses against the pulse intensity. **(E)** Comparisons of latencies of APS evoked by cathodic and biphasic pulses with pulse intensities of 0.3, 0.5, 1.0, and 1.5 mA (^**^p < 0.01, ^***^p < 0.001, paired *t*-test, *n* = 12).

Area of APS can represent the amount of neuronal firing to simultaneously respond to a stimulus (Andersen et al., 1971; Theoret et al., 1984; Varona et al., 2000). With an intensity of 1.0 mA, the mean area of APS evoked by a biphasic pulse was significantly greater than the mean area of APS evoked by a cathodic pulse (Area_┼_ = 13.35 ± 0.53 mV.ms vs. Area_┬_ = 12.32 ± 0.58 mV.ms, *n* = 12, paired *t*-test, *p* < 0.01; Figure 1C-3). The result further indicated that the anodic phase of a biphasic pulse can facilitate, rather than prevent, the firing of neuronal population.

A pulse with a different intensity can activate neurons in a differently sized region, thereby generating an APS with a different area. When the pulse intensity (defined as variable *x*) increased from 0.1 to 2.0 mA with a step of 0.1 mA, the mean areas of APS (defined as variable *y*) evoked by the three types of pulses all increased monotonically, with a change fitted well by an exponential curve *y* = *a* - *b*e^–^*^x^*^/^*^τ^* (*R*^2^ > 0.99, Figure 1D). According to the theory of a first-order linear system characterized by an exponential equation, triple of the coefficient τ is the *x*-value at which the *y* reaches ∼95% of the steady-state value *a* (i.e., “saturation” value). Here we termed the pulse intensity *x* = 3τ as the beginning of saturation. The 3τ of Area_┬_, Area_┼_, and Area_┴_ was approximately 0.8, 1.2, and 2.3 mA, respectively. In addition, the steady-state values of Area_┼_ and Area_┴_ (*a* = 14.4 and 14.2 mV.ms) were greater than Area_┬_ (*a* = 12.6 mV.ms). When the pulse intensity was 0.1 mA, the mean Area_┴_ was only ∼13% of Area_┬_ and ∼16% of Area_┼_. However, when the pulse intensity increased to above 1.8 mA, the mean Area_┴_ was even greater than Area_┬_. With the increase of Area_┴_ along with the increase of pulse intensity, Area_┼_ changed from being significantly smaller than Area_┬_ (e.g., at 0.3 mA, 8.58 ± 0.52 vs. 8.88 ± 0.52 mV.ms) to significantly greater than Area_┬_ (e.g., at 1.0 mA, 13.35 ± 0.53 vs. 12.32 ± 0.58 mV.ms, paired *t*-test, *p* < 0.01, *n* = 12; Figures 1E,F).

With the pulse intensity increasing from 0.1 to 2.0 mA, the mean latencies of APS evoked by the three types of pulses (i.e., Lat_┬_, Lat_┼_, and Lat_┴_) also changed. Lat_┴_ and Lat_┼_ decreased monotonically, while Lat_┬_ did not changed markedly (Figure 1G). The mean Lat_┬_ was always significantly longer than Lat_┼_, and the difference increased with the increase of pulse intensity. Examples of statistical comparisons for 0.3, 0.5, and 1.0 mA are shown in Figure 1H.

The above-mentioned results indicated that an anodic pulse can activate neurons, and its activation effect can even exceed a cathodic pulse with a relatively strong pulse intensity (e.g., 2.0 mA). In addition, even when the activation effect of an anodic pulse is far weaker than that of a cathodic pulse, such as at 1.0-mA stimulation, the anodic phase can also facilitate a biphasic pulse to activate more neurons to fire (Figures 1C,F).

With a weak pulse intensity, such as 0.3 mA, the anodic phase of a biphasic pulse did weaken the activation of the preceding cathodic pulse, thereby resulting in less firing of neurons induced by a biphasic pulse than a cathodic pulse (Figure 1F). Nevertheless, previous studies have shown that a short IPG may eliminate the reversal effect of the anodic phase (Merrill et al., 2005). Therefore, to further confirm the influence of anodic phase, we next set an IPG of 100 μs between the two phases of biphasic pulses and termed this type of pulse as paired pulse (Figure 2A). The length of IPG was defined as the interval from the end of the preceding cathodic phase to the onset of the subsequent anodic phase. Thus, a biphasic pulse is equivalent to a paired pulse with an IPG = 0.

The comparisons of APS areas evoked by the three types of pulses—biphasic pulse (Area_┼_), paired pulse (Area_┼*IPG*_), and monophasic cathodic pulse (Area_┬_)—showed that when the pulse intensity was 0.3 mA, the mean Area_┼__*IPG*_ was significantly greater than the mean Area_┼_ (9.09 ± 0.56 vs. 8.72 ± 0.51 mV.ms, two-way ANOVA, *post hoc* LSD test, *p* < 0.001, *n* = 7; Figure 2B). However, the mean Area_┼__*IPG*_ was not significantly greater than the mean Area_┬_ (9.09 ± 0.56 vs. 9.04 ± 0.56 mV.ms, *post hoc* LSD test, *p* = 0.43, *n* = 7). The result indicated that a short IPG of 100 μs can weaken the effect of anodic phase to prevent neuronal firing. Not surprisingly, when the pulse intensity was increased to 0.5 and 1.0 mA, Area_┼__*IPG*_ was significantly greater than both Area_┼_ and Area_┬_ (Figures 2C,D).

The above-mentioned results were obtained from monopolar stimulations. Because bipolar stimulations have been commonly used in clinical brain stimulations to limit action ranges, we next investigated the effects of different types of pulses in a bipolar stimulation mode.

### The Anodic Phase of Biphasic Pulses Can Facilitate the Activation of Neuronal Population in Bipolar Stimulations

In the configuration of bipolar stimulations (Figure 3A), the pulse current flowed between the inner pole and the outer pole of the stimulation electrode and acted on the alveus fiber close to the inner pole.

When the pulse intensity increased from 0.1 to 2.0 mA, the mean areas of APS evoked by the three types of pulses all increased monotonically with exponential curve fittings (*R*^2^ > 0.99; Figure 3B). The pulse intensities (3τ) for the beginning of saturation of Area_┬_, Area_┼_, and Area_┴_ were 0.9, 1.4, and 2.3 mA, respectively. In addition, the steady-state values of Area_┼_ and Area_┴_ (*a* = 13.3 and 12.8 mV.ms) were greater than Area_┬_ (*a* = 12.0 mV.ms). These results were similar to the corresponding data of monopolar stimulations (Figure 3B vs. Figure 1D). With the increase of Area_┴_ along with the increase of pulse intensity, Area_┼_ changed from significantly smaller than Area_┬_ (e.g., at 0.3 mA, 7.46 ± 0.64 vs. 7.94 ± 0.59 mV.ms) to significantly greater than Area_┬_ (e.g., at 1.5 mA, 12.91 ± 0.55 vs. 11.88 ± 0.53 mV.ms, paired *t*-test, *p* < 0.01, *n* = 12; Figures 3B,C).

With the pulse intensity increasing from 0.1 to 2.0 mA, the latency of APS evoked by the anodic pulse (Lat_┴_) and biphasic pulse (Lat_┼_) decreased monotonically, while the latency of APS evoked by the cathodic pulse (Lat_┬_) did not change markedly (Figure 3D). The mean Lat_┬_ was always significantly longer than Lat_┼_, and the difference increased with the increase of pulse intensity. Examples of statistical comparisons for 0.3, 0.5, 1.0, and 1.5 mA are shown in Figure 3E.

**FIGURE 4:**
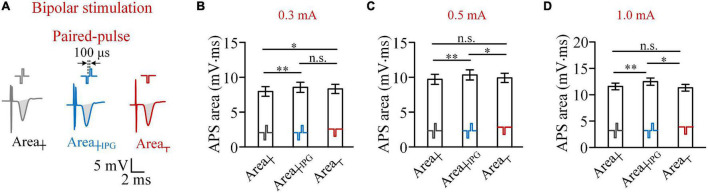
Comparisons of areas of antidromically evoked population spike (APS) evoked by biphasic pulses, paired pulses, and cathodic pulses in bipolar stimulations. **(A)** Examples of APS evoked by the three types of pulses. **(B–D)** Comparisons of mean areas of APS evoked by the three types of pulses with pulse intensities of 0.3, 0.5 and 1.0 mA, respectively (*p < 0.05, ^**^p < 0.01; n.s., not significant, two-way ANOVA, *post hoc* least significant difference test, *n* = 7).

When an IPG of 100 μs was inserted between the two phases of biphasic pulse to form a paired pulse (Figure 4A), with a pulse intensity of 0.3 mA, the Area_┼__*IPG*_ was not smaller than the Area_┬_ (8.57 ± 0.70 vs. 8.32 ± 0.64 mV.ms; two-way ANOVA, *post hoc* LSD test, *p* = 0.08, *n* = 7). Meanwhile, the Area_┼_ was significantly smaller than the Area_┬_ (8.02 ± 0.68 vs. 8.32 ± 0.64 mV.ms; *post hoc* LSD test, *p* < 0.05, *n* = 7; Figure 4B) due to the reverse effect of the anodic phase. When the pulse intensity increased to 0.5 mA or above, the Area_┼__*IPG*_ was significantly greater than both Area_┼_ and Area_┬_ (two-way ANOVA, *post hoc* LSD test, *p* < 0.05, *n* = 7; Figures 4C,D). The results indicated that a short IPG can eliminate the reverse effect of the anodic phase and even enable the anodic phase to facilitate the activation of neurons.

So far, the data from rat experiments *in vivo* suggested that a biphasic pulse can activate more neurons than a monophasic cathodic pulse in both monopolar and bipolar stimulations. It was intriguing that the opposite phase (i.e., the subsequent anodic phase) did not counteract but rather facilitated the effect of the preceding cathodic phase. We next utilized a computational model of myelinated axons to reveal possible underlying mechanisms that generate the results.

### Computational Modeling of the Activation of Different Types of Pulses on Axons

**FIGURE 5:**
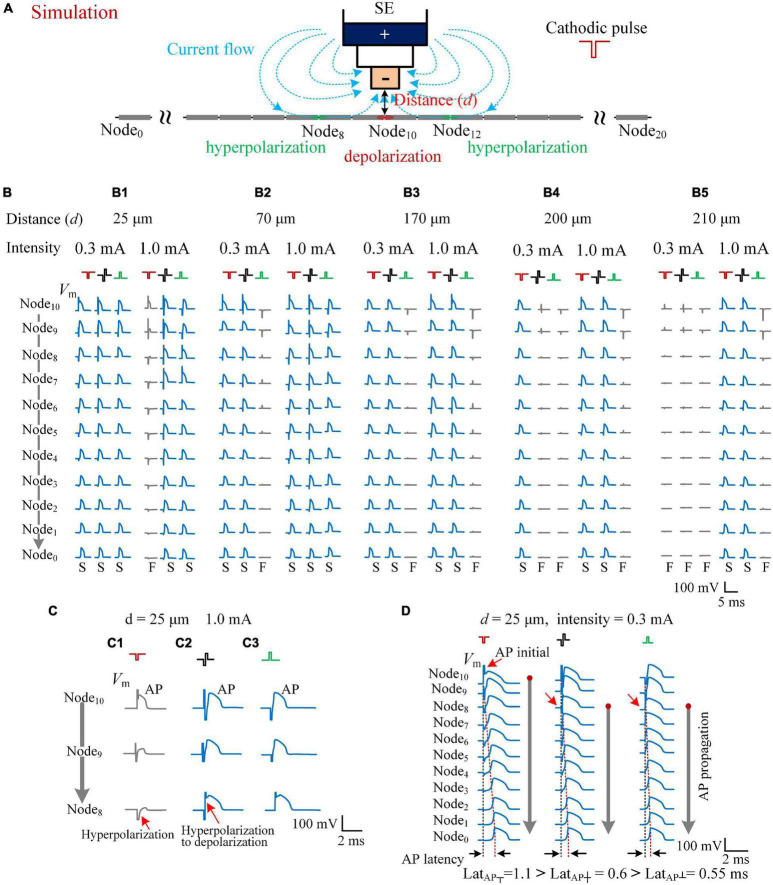
Simulating the effects of cathodic, biphasic, and anodic pulses on axons in different distances. **(A)** Schematic diagram of the computational model consisting of a stimulation electrode (SE) and an axon with 21 nodes. When a cathodic pulse was applied, the current flowed from the outer pole to the inner pole of SE (denoted by blue dotted lines with arrows). The center node (Node_10_) of the axon was depolarized, and the flanking regions (e.g., Node_8_ and Node_12_) were hyperpolarized. **(B)** The membrane potentials (*V*_*m*_) evoked along Node_10_–Node_0_ of the five axons in different distances to SE (*d* = 25, 70, 170, 200, and 210 μm) by the three types of pulses with a pulse intensity of 0.3 and 1.0 mA. The *V*_*m*_ waveforms in blue with a letter “S” in the bottom denote that the applied pulse successfully induced an action potential (AP), while the *V*_*m*_ waveforms in gray with a letter “F” in the bottom denote that the applied pulse failed to induce a propagatable AP. **(C)** The axon in the distance 25 μm from the SE was activated by both biphasic and anodic pulses successfully, but not by the cathodic pulse with an identical pulse intensity of 1.0 mA because of the hyperpolarization block at Node_9_ and Node_8_. **(D)** The *V*_*m*_ waveforms evoked along Node_10_–Node_0_ of the axon, at a distance of 25 μm, showing different initial sites (red arrows) of AP evoked by the three types of pulses with a pulse intensity of 0.3 mA. The latencies of AP (Lat_*AP*_) are denoted in the bottom.

In the computational model similar to previous reports (Guo et al., 2018), stimulation pulses were delivered in the bipolar mode through a concentric bipolar electrode with the same structures and size as that used in the rat experiments. The axons of mimicked alveus were located right below the electrode, with their central node (Node_10_) having a vertical distance (termed as *d*) from the bottom center of the electrode in the range of *d* = 25–210 μm (Figure 5A). When a cathodic pulse was applied, the electrical current flowed inwards the axonal membrane at the two flanking regions (e.g., Node_8_ and Node_12_) and flowed outwards the axonal membrane at the central region (i.e., Node_10_) closest to the inner pole of the electrode. The outward current depolarized the membrane and was able to initiate an AP at Node_10_ (Figure 5A), while the inward current hyperpolarized the membrane at the two flanking regions. On the contrary, when an anodic pulse was applied, the sites of depolarization and hyperpolarization were reversed. The flanking regions (Node_8_ and Node_12_) were depolarized to initiate an AP.

The activation degree of each axon was related with the pulse intensity, the distance *d*, and the type of applied pulse—for example, with an identical pulse intensity of 0.3 mA, all of the three types of pulses—cathodic pulse, biphasic pulse, and anodic pulse—were able to activate the axon at the distance *d* = 25 μm to generate an AP to propagate successfully to both ends of the axon (e.g., Node_0_). However, when the pulse intensity increased to 1.0 mA, the cathodic pulse failed to generate a propagable AP, but the other two pulses succeeded (Figure 5B-1). For an axon at a longer distance *d* = 70 μm, both the cathodic pulse and the biphasic pulse successfully generated a propagable AP with either 0.3 or 1.0 mA intensity, but the anodic pulse failed with 0.3-mA intensity (Figure 5B-2). For an axon at a farther distance *d* = 170 μm, even with 1.0-mA intensity, the anodic pulse also failed to generate a propagable AP, let alone at *d* = 200 and 210 μm (Figures 5B-2–5). For an axon at *d* = 200 μm, the biphasic pulse also failed with 0.3-mA intensity (Figure 5B-4). Finally, with the distance further increased to 210 μm, all the three types of pulses failed to generate a propagable AP with the relatively weak intensity of 0.3 mA. Nevertheless, with an intensity of 1.0 mA, the cathodic pulse and the biphasic pulse were still able to elicit an AP (Figure 5B-5).

Except the distance of 25 μm, at the distance of 70–210 μm, the modeling results of axonal responses followed the efficiency order of activation: cathodic pulse > biphasic pulse > anodic pulse (Figures 5B-2–5), which conformed to the common sense of extracellular stimulations. The paradoxical situation was that, at a close distance of 25 μm, with a relatively strong intensity of 1.0 mA, the activation ability of cathodic pulse was lowest (Figure 5B-1). Our modeling data showed that, at this situation, the failure of axonal activation by the cathodic pulse was caused by hyperpolarization block in the flanking regions (Figure 5C). The cathodic pulse did induce an AP at the central Node_10_ of the axon, but the AP failed to propagate out because the membrane of the flanking regions (e.g., Node_8_) was hyperpolarized substantially and prevented the propagation of AP (Figure 5C-1). When a biphasic pulse was applied, the subsequent anodic phase generated a reversal transmembrane current to rapidly reverse the membrane potential in the flanking regions from hyperpolarization to depolarization, thereby facilitating the propagation of AP (Figure 5C-2). For an anodic pulse, the membrane of the flanking regions was depolarized sufficiently to an initial AP due to the activation effect of the virtual cathode (Figure 5C-3).

In addition, in the situation of a cathodic pulse applied, the hyperpolarization at the flanking regions also slowed down the propagation of AP, which can explain the longer latency of APS evoked by cathodic pulses (Lat_┬_) in rat experiments. Taking the axon at *d* = 25 μm as an example (Figure 5D), although all the three types of pulses were able to generate an AP with a pulse intensity of 0.3 mA, the initial sites of AP were different (Figure 5D, red arrow). With a cathodic pulse applied, the AP initiated at the center of the axon (Node_10_) and then propagated slowly through the flanking regions that were under hyperpolarization by the cathodic pulse itself. On the contrary, with a biphasic pulse or an anodic pulse applied, the AP initiated at the flank of the axon (Node_8_) and then propagated fast through the farther flanking regions that were under depolarization by the anodic phase of the biphasic pulse or by the anodic pulse itself. Consequently, the three types of pulse stimulations resulted in differences in “AP latency” (Lat_*AP*_). In computational modeling, the Lat_*AP*_ was defined as the time distance between the onset of applied pulse and the peak of AP that finally appeared at the end of the axon (Node_0_). Thus, the cathodic pulse generated the longest Lat_*AP*_ (1.1 ms), and the anodic pulse generated the shortest one (0.55 ms) (Figure 5D, bottom). The order was consistent with the results observed in the rat experiments (Figures 1G, 3D).

These results of computational modeling showed that, although the cathodic pulse has the strongest depolarization effect in extracellular stimulations, the activation region of a cathodic pulse can be smaller than that of a biphasic pulse—for example, with the intensity of 1.0 mA, among the five axons in Figure 5B, one axon was not activated by the cathodic pulse, but all of the axons were activated by the biphasic pulse. The anodic phase of a biphasic pulse can relieve the hyperpolarization block generated by the preceding cathodic phase, thereby facilitating the activation of the biphasic pulse. In addition, the fact that the latency of AP induced by a biphasic pulse was shorter than that induced by a cathodic pulse further confirms the mechanism of the anodic phase to facilitate the generation and propagation of AP along the axon.

## Discussion

The novel finding of this study is that, with a sufficient pulse intensity, the anodic phase in a biphasic pulse stimulation can facilitate rather than prevent the neuronal firing in both monopolar and bipolar modes in axonal stimulations. The reason may be that the subsequent anodic phase can relieve the hyperpolarization block in the flanking regions generated by the preceding cathodic phase, thereby increasing the activation efficiency of the biphasic pulse. To our knowledge, this is the first report that reveal the interesting phenomenon. The underlying mechanisms and the implications of the finding are discussed below.

### Biphasic Pulse Can Be More Efficient Than Cathodic Pulse to Activate Axons

In our rat experiments *in vivo*, the stimulation pulses were applied on the alveus, a layer of axons of the pyramidal cells—the principal neurons of the hippocampal CA1 region. Before reaching a “saturation” state, the evoked neuronal firing (represented by the APS area) increased with the increase of pulse intensity, indicating an enlarging volume of activated axons by the pulse (Figures 1D, 3B). In theory, the ability to activate axons should be greatest for the cathodic pulse among the three types of pulses: cathodic pulse, biphasic pulse, and anodic pulse (Miller et al., 2001; Merrill et al., 2005). However, the cathodic pulse failed to induce the maximum amount of neuronal firing due to an earlier saturation during the increase of pulse intensity (see the exponential curve fitting in Figures 1D, 3B). This firing saturation induced by cathodic pulses may be caused by a balance between a decrease of firing by the hyperpolarization block in the region close to the electrode and an increase of firing in the extending region of the activation edge (Figures 5B-1–5). With the addition of anodic phase in biphasic pulses, the hyperpolarization block induced by the preceding cathodic phase can be eliminated. Therefore, the neuronal firing can increase further with the increase of pulse intensity. In the situation without the effect of hyperpolarization block, even the neuronal firing induced by an anodic pulse can continuously increase to exceed the neuronal firing induced by a cathodic pulse with a relatively high intensity. Our modeling results verified these inferences. Previous reports have also shown that hyperpolarization blocks appear at the flanking regions of stimulated axons in cathodic stimulations (Tai et al., 2009; Van de Steene et al., 2020).

In addition, our rat experiments showed a shorter latency of neuronal firing induced by a biphasic pulse than by a cathodic pulse. This type of latency differences has also been reported in previous studies in the stimulations of cat auditory (Miller et al., 2001). Our modeling results suggest that the hyperpolarization in the flanking regions may delay the propagation of action potential, thereby generating a longer latency in the firing induced by cathodic pulses (Figure 5D).

Finally, the facilitation of anodic phase in biphasic-pulse stimulations appeared in both monopolar and bipolar modes. It may be argued that, in bipolar stimulation, the polarities of electrical fields in the regions around the two poles were opposite (Figures 3A, 5A). The firing induced by an anodic pulse or an anodic phase could be caused by the activation of axons close to the outer pole of the stimulation electrode where the “anodic pulse”—defined according to the inner pole—actually functioned as a cathodic pulse. However, the situation could not appear in our experiment preparations. The alveus is a thin layer of axon fibers. With a pulse intensity smaller than 0.5 mA (Figure 3B), a much greater amount of firing was induced by a cathodic pulse (or a biphasic pulse) than by an anodic pulse. This fact ensured that the layer of alveus was located closely to the inner pole, not to the outer pole of the stimulation electrode. In addition, similar results obtained in the monopolar mode only using the inner pole (Figure 1) confirmed that the facilitation of anodic phase was not due to the action of an opposite pole.

Taken together, a hyperpolarization block may be generated when a cathodic pulse with a high intensity is applied to extend the activation region of neural stimulations. Under this situation, the anodic phase of biphasic pulse can eliminate the hyperpolarization block and increase the activation efficiency of the cathodic phase.

### Implications and Limitations

Both monopolar and bipolar stimulations have been utilized in the clinic for neural stimulations, such as deep brain stimulation (DBS) (Montgomery, 2010; Almeida et al., 2016; Picillo et al., 2016). Our findings in this study have implications for guiding these stimulations.

The findings provide new clues for the design of stimulus waveforms. To decrease the reversal effects of the anodic phase in charge-balanced stimulations, conventional DBS pulses use asymmetric biphasic pulses, including a low-intensity, long-width anodic phase (Foutz and McIntyre, 2010; Akbar et al., 2016; Almeida et al., 2017). Various stimulation waveforms have been proposed for reducing energy consumption or improving the activation efficiency, including rectangular and non-rectangular stimulus waveforms (e.g., exponential, triangular, Gaussian, and sinusoidal stimulus pulse shapes) (Foutz and McIntyre, 2010; Jezernik et al., 2010; Wongsarnpigoon and Grill, 2010). Nevertheless, some clinical trials have shown that the simple square biphasic pulses are also safe and effective in treating tremor symptom, Parkinson’s disease, and dystonia (Almeida et al., 2017; De Jesus et al., 2018, 2019), but the underlying mechanisms have not been investigated yet. The present study showed that the anodic phase (or anodic pulse) of a symmetrical square biphasic pulse can play a facilitation role, in the meantime, to balance the charges. Therefore, a design of asymmetrical biphasic pulse may not be necessary under some circumstances.

The study also provides clues for the design of small electrodes. To limit damages to neural tissues, the implanted electrodes should be as small as possible. The electrical field radiated from a small electrode is inhomogeneous. When the electrical field far away is strong enough to activate neurons in a required large region, the stronger field in the closer vicinity of the electrode could have generated a hyperpolarization block on the neurons there. The release of a hyperpolarization block by the anodic phase of a biphasic pulse may facilitate the activation of a small electrode to cover a region large enough to meet the need of stimulation therapies.

This study investigated the neuronal responses to axonal stimulation. Activations of axons may play an important role in brain stimulations because an axon is the most sensitive structure to pulse stimulations in a neuron and because the firing in axonal fibers can propagate out to modulate neuronal activity in large regions (Grill et al., 2004; Johnson et al., 2008; Shah et al., 2010). Even if the stimulation site is located at the nucleus, the firing may also be initiated from the axons (McIntyre et al., 2004). Nevertheless, studies of neuronal responses to stimulations near cell bodies are needed to further verify the results observed in the present study of axonal stimulations. In addition, the amount of neuronal firing responding to single pulses was used to evaluate the activation ability of different types of pulses. In neural therapies, such as DBS, vagus nerve stimulation, and spinal cord stimulation, continuous pulse sequences of high-frequency stimulations have been used (Fisher and Velasco, 2014; Edwards et al., 2017). The effect of anodic phases in those sequences also needs further studies. Moreover, here we took the advantage of a high soma density and a clear lamellar organization in the structures of hippocampal regions to record the APS potentials to evaluate the neuronal firing. In other brain regions without the advantage of APS usage, it should be a challenge to measure the amount of neuronal firing on responding to a pulse by recording action potentials from individual neurons, especially for *in vivo* animal experiments. Although the present findings may be extrapolated to axonal stimulations in other brain regions, verifications are needed. Therefore, further investigations utilizing more techniques are expected to verify the universality of the findings.

## Conclusion

Both rat experiments and computational modeling showed a facilitation rather than an attenuation of the anodic phase in a biphasic pulse to activate neurons in axonal stimulations. The results provide important information for waveform designs of stimulation pulses and for size designs of electrodes in neural stimulations.

## Data Availability Statement

The original contributions presented in the study are included in the article/supplementary material, further inquiries can be directed to the corresponding author/s.

## Ethics Statement

The animal study was reviewed and approved by the Institutional Animal Care and Ethics Committee, Zhejiang University.

## Author Contributions

ZF and LZ designed the study, interpreted the results, and wrote the manuscript. LZ, YY, and YH performed the animal experiments. LZ and YX analyzed the experimental data. LZ created the computational model and performed the modelings. All authors approved the final version for submission.

## Conflict of Interest

The authors declare that the research was conducted in the absence of any commercial or financial relationships that could be construed as a potential conflict of interest.

## Publisher’s Note

All claims expressed in this article are solely those of the authors and do not necessarily represent those of their affiliated organizations, or those of the publisher, the editors and the reviewers. Any product that may be evaluated in this article, or claim that may be made by its manufacturer, is not guaranteed or endorsed by the publisher.
